# Supplementary figures and images for: Oxidized low-density lipoprotein potentiates angiotensin II-induced Gq activation through the AT1-LOX1 receptor complex
Source: eLife. 2025 Mar 25;13:RP98766. doi: 10.7554/eLife.98766 (PMC11936421; doi:10.7554/eLife.98766)

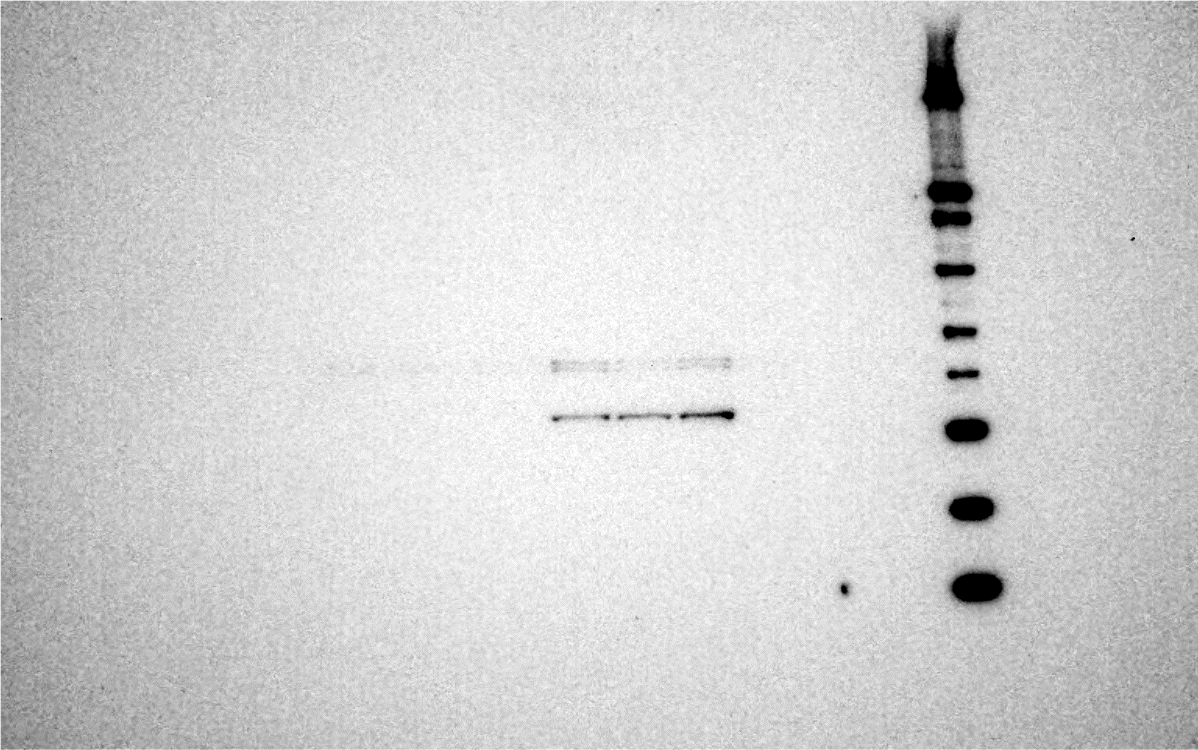

Supplement: Figure 5—source data 2. — Original blots for western blot analysis displayed in Figure 5. [file elife-98766-fig5-data2.zip › 5g a-SMA.TIFF]

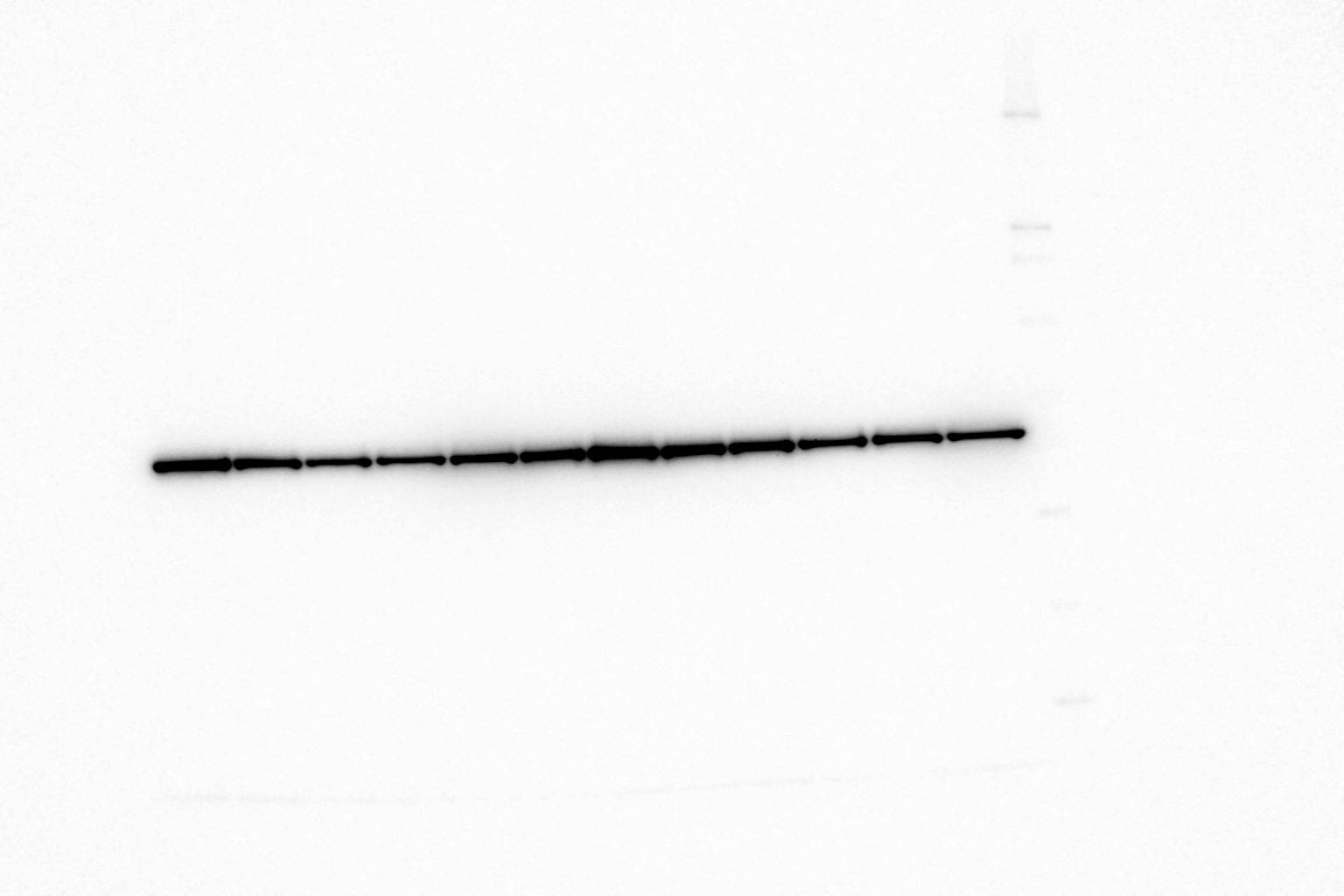

Supplement: Figure 5—source data 2. — Original blots for western blot analysis displayed in Figure 5. [file elife-98766-fig5-data2.zip › 5g a-tubulin.TIF]

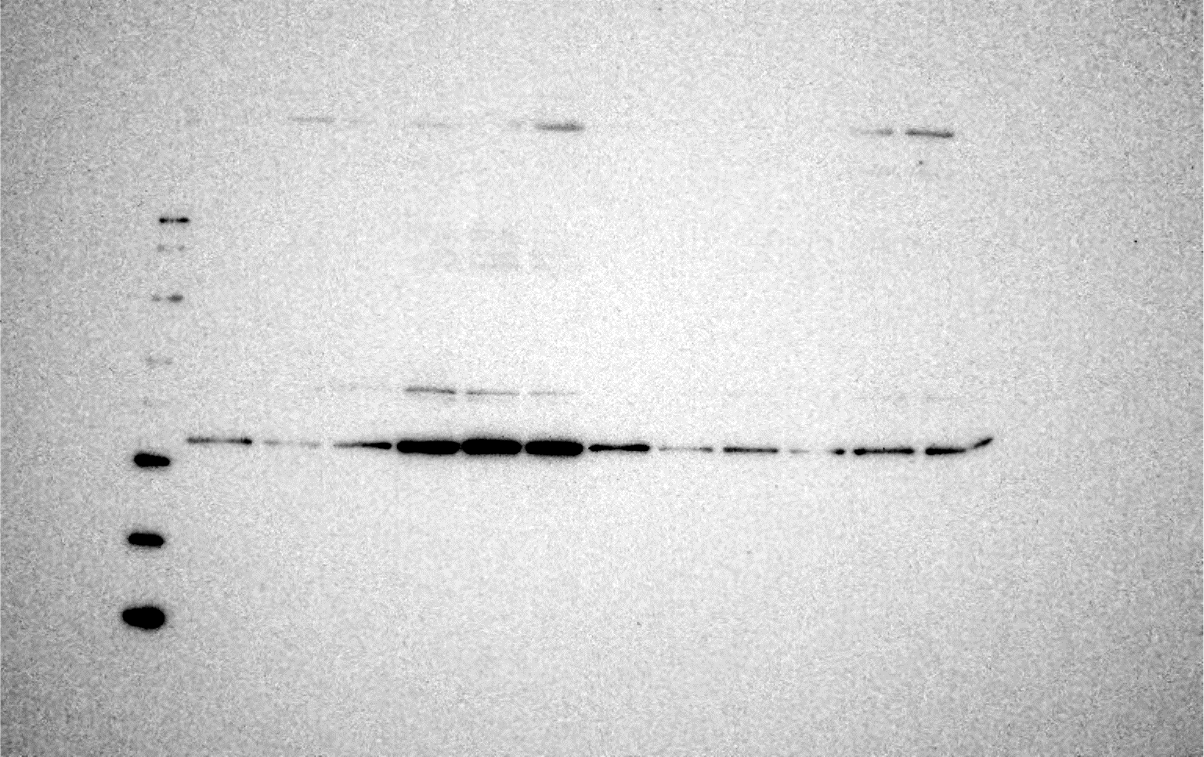

Supplement: Figure 5—source data 2. — Original blots for western blot analysis displayed in Figure 5. [file elife-98766-fig5-data2.zip › 5h a-SMA.TIFF]

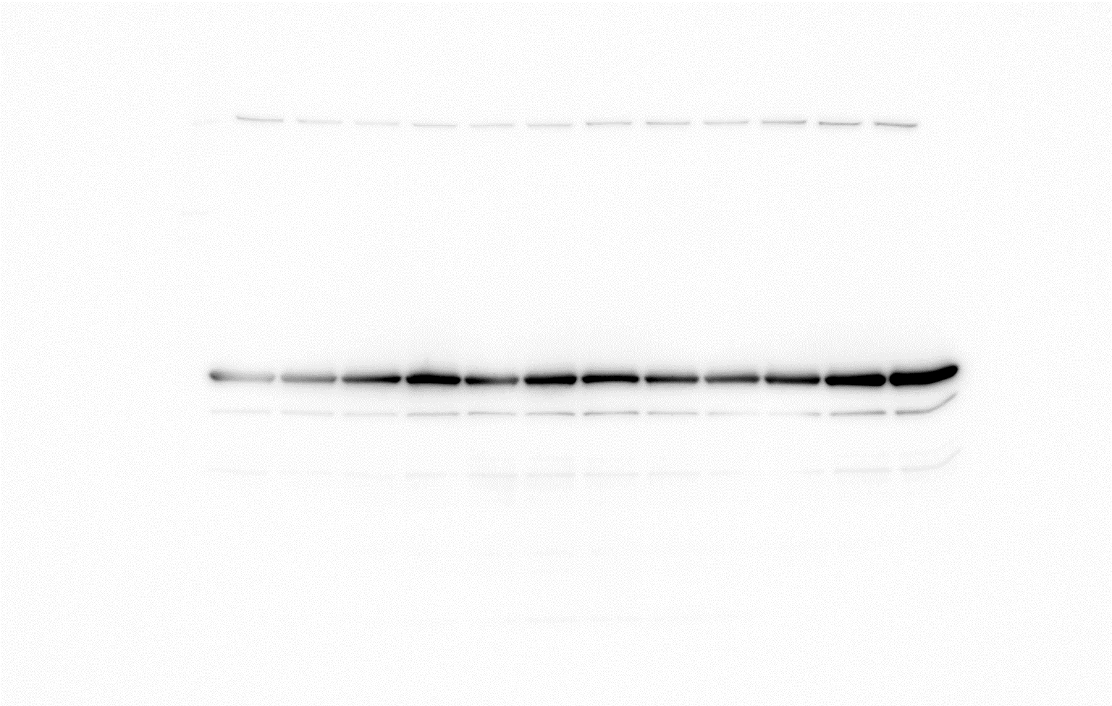

Supplement: Figure 5—source data 2. — Original blots for western blot analysis displayed in Figure 5. [file elife-98766-fig5-data2.zip › 5h a-tubulin.TIFF]

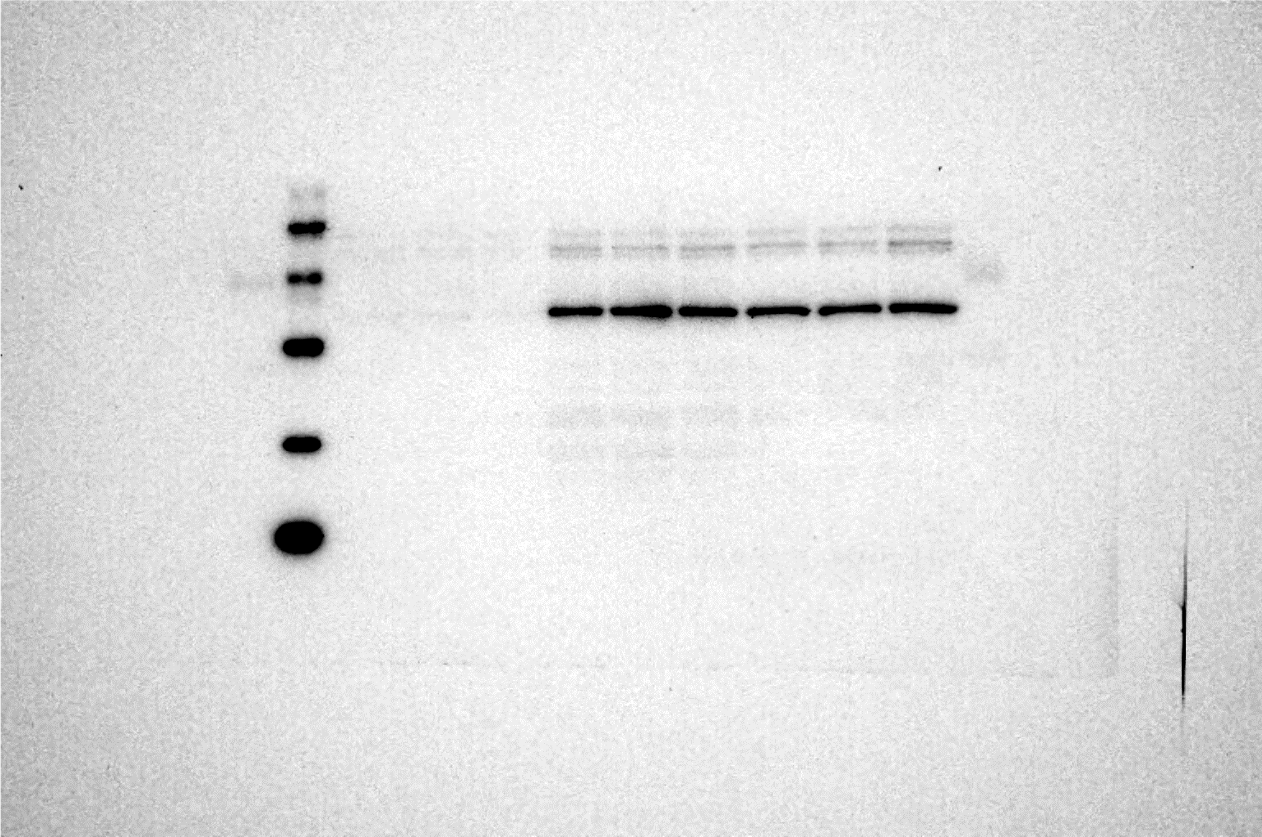

Supplement: Figure 5—source data 2. — Original blots for western blot analysis displayed in Figure 5. [file elife-98766-fig5-data2.zip › 5a a-SMA.TIFF]

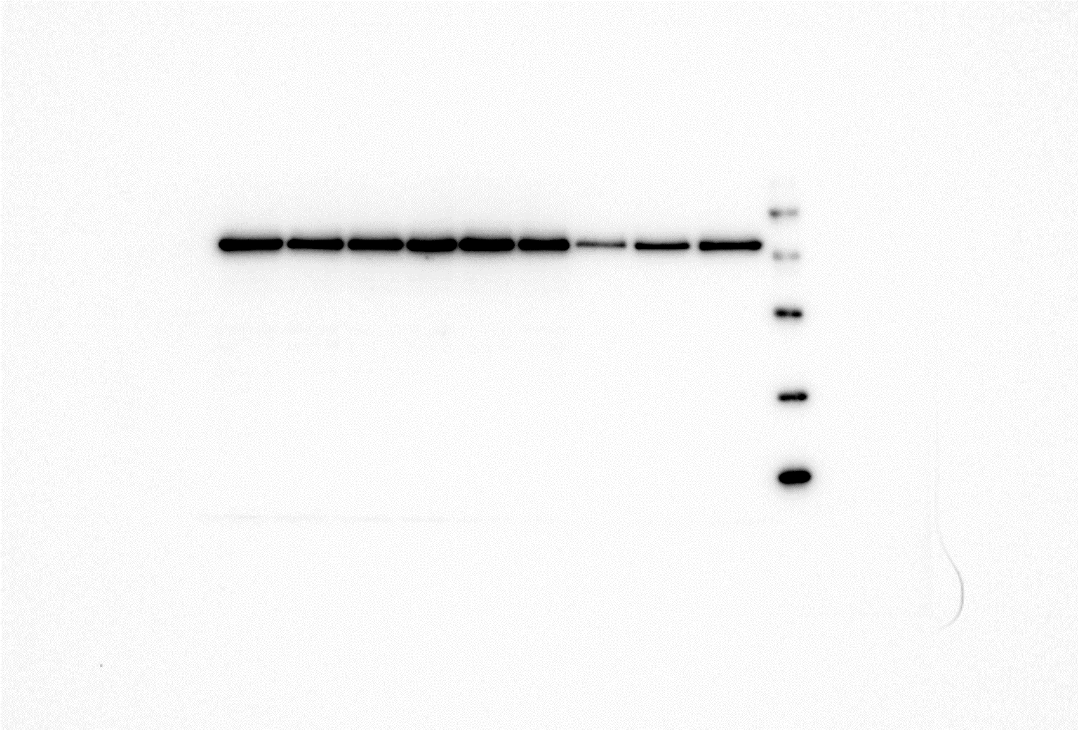

Supplement: Figure 5—source data 2. — Original blots for western blot analysis displayed in Figure 5. [file elife-98766-fig5-data2.zip › 5a a-tubulin.TIFF]

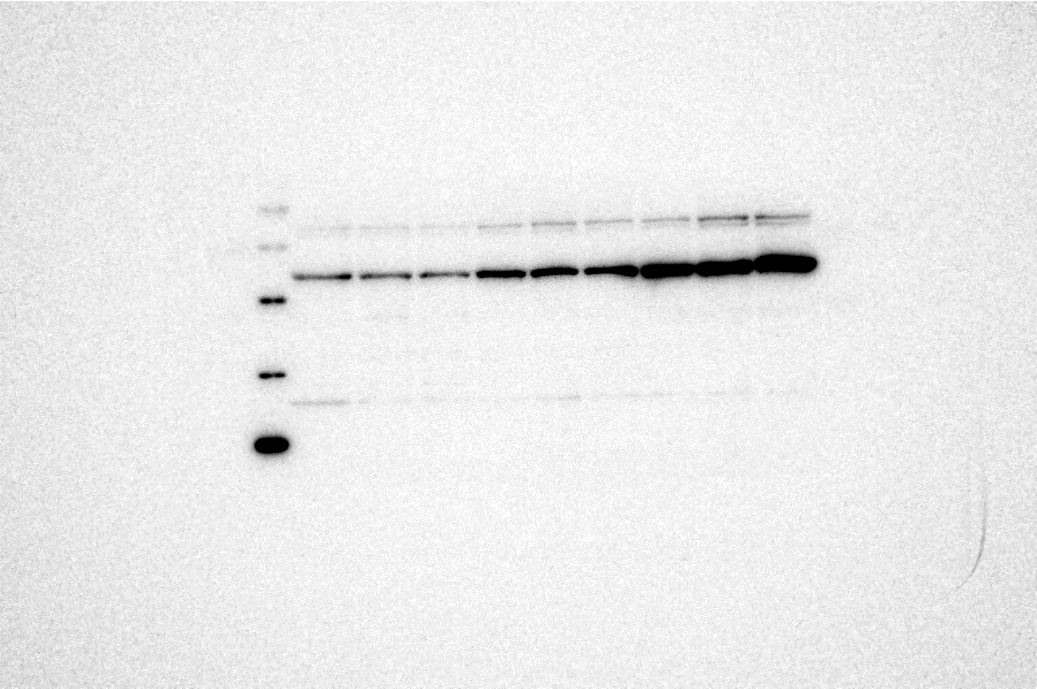

Supplement: Figure 5—source data 2. — Original blots for western blot analysis displayed in Figure 5. [file elife-98766-fig5-data2.zip › 5b a-SMA.TIFF]

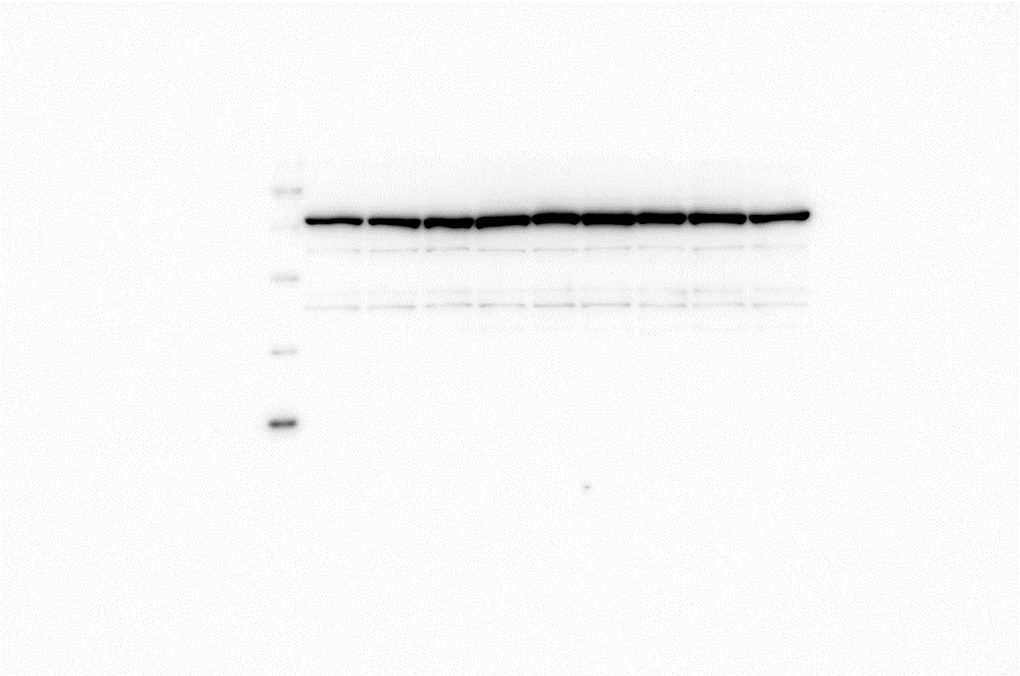

Supplement: Figure 5—source data 2. — Original blots for western blot analysis displayed in Figure 5. [file elife-98766-fig5-data2.zip › 5b a-tubulin.TIFF]

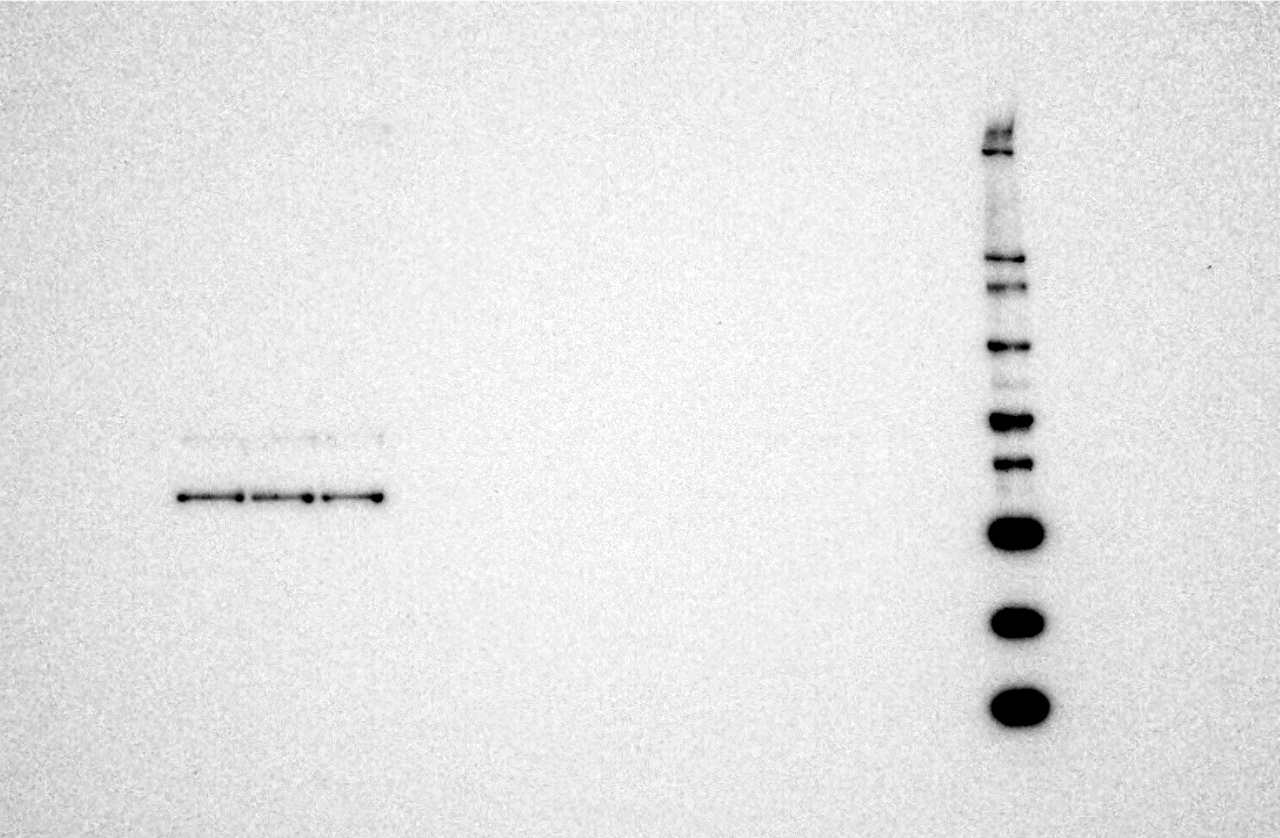

Supplement: Figure 5—source data 2. — Original blots for western blot analysis displayed in Figure 5. [file elife-98766-fig5-data2.zip › 5c a-SMA.TIFF]

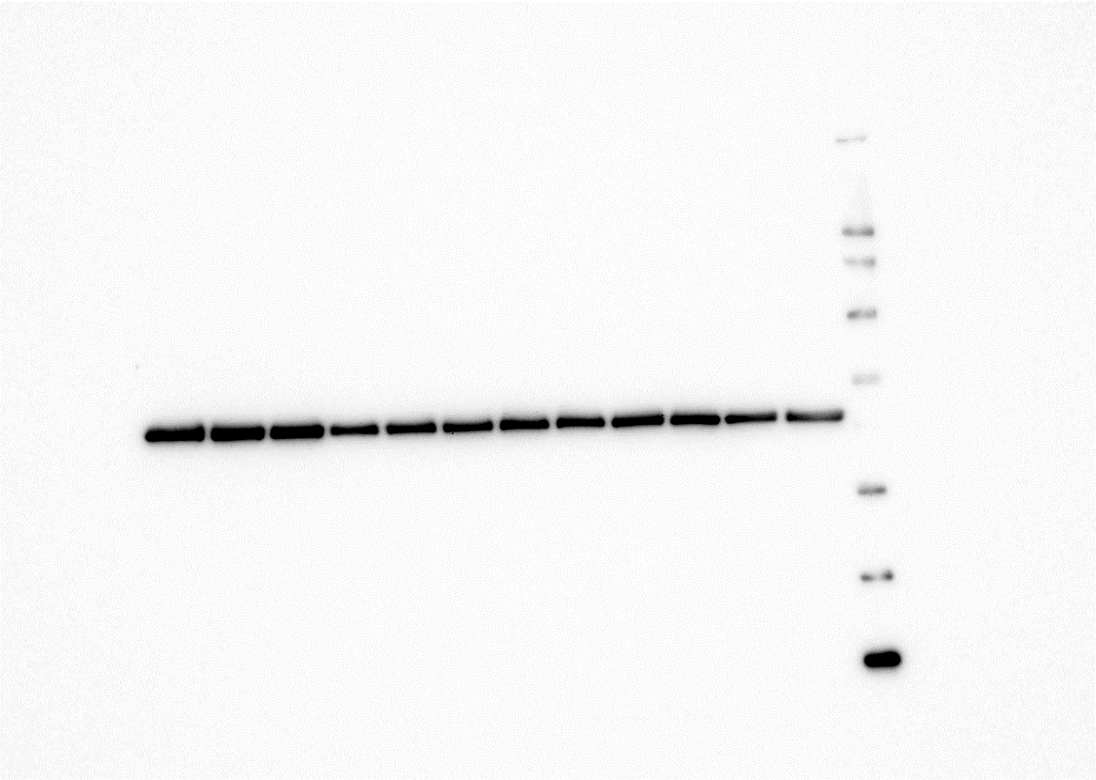

Supplement: Figure 5—source data 2. — Original blots for western blot analysis displayed in Figure 5. [file elife-98766-fig5-data2.zip › 5c a-tubulin.TIFF]

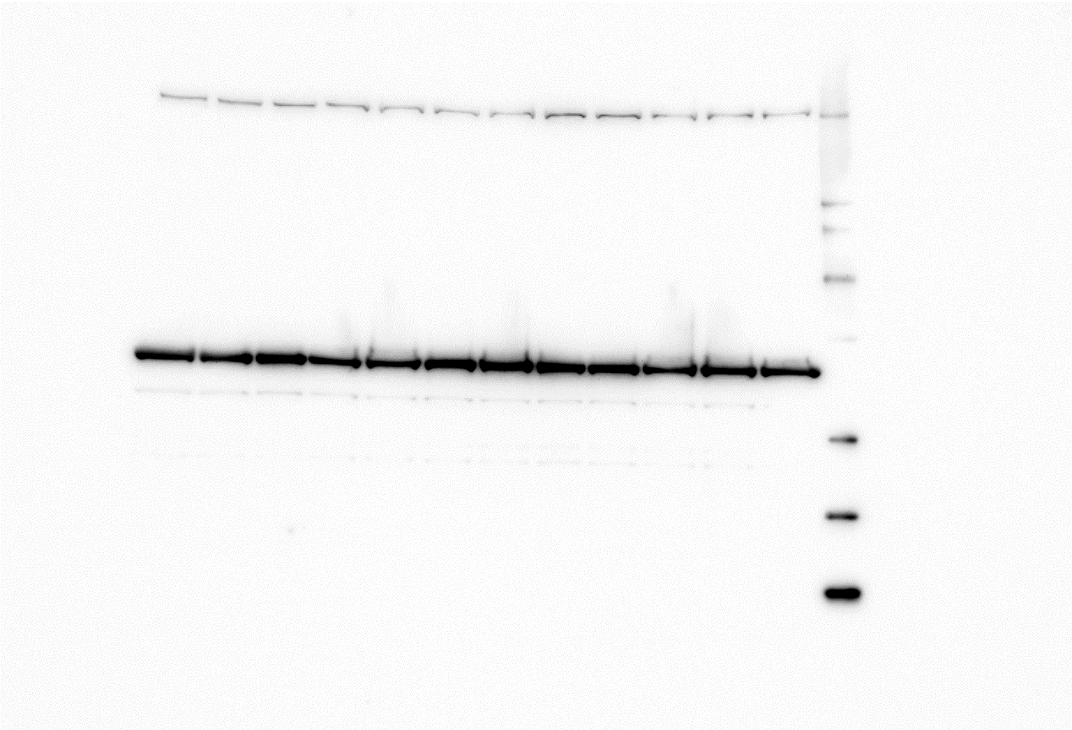

Supplement: Figure 5—source data 2. — Original blots for western blot analysis displayed in Figure 5. [file elife-98766-fig5-data2.zip › 5d a-tubulin.TIFF]

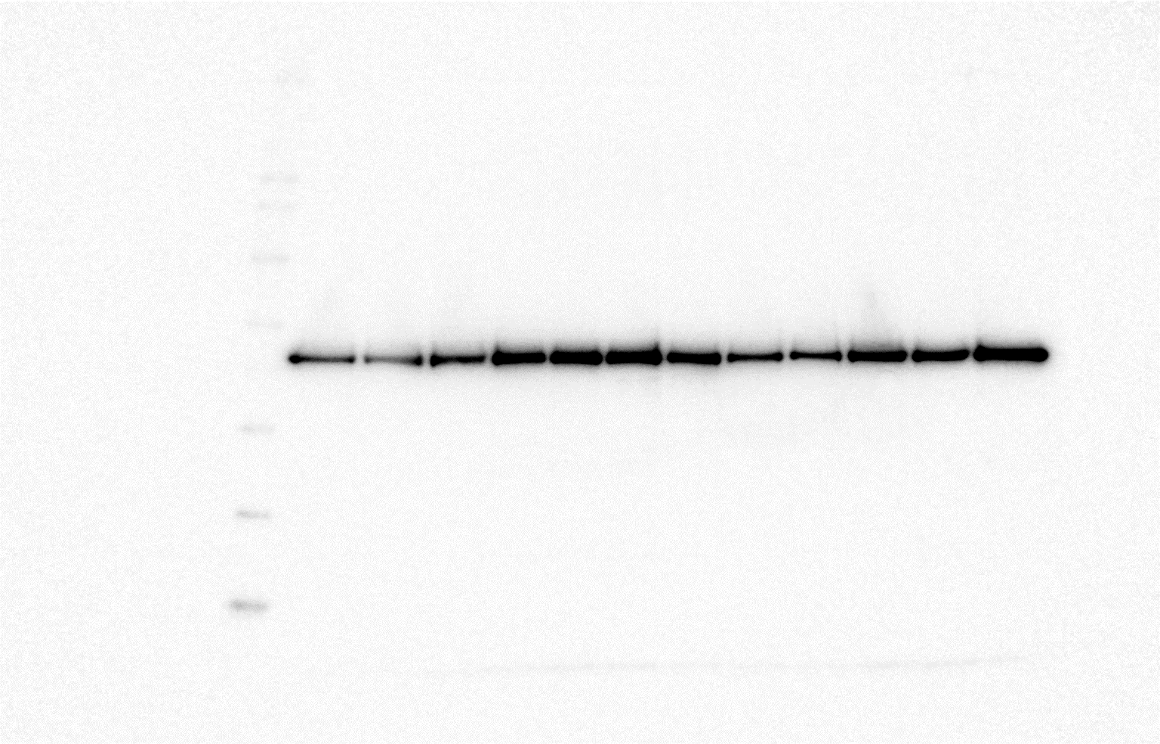

Supplement: Figure 5—source data 2. — Original blots for western blot analysis displayed in Figure 5. [file elife-98766-fig5-data2.zip › 5e a-tubulin.TIFF]

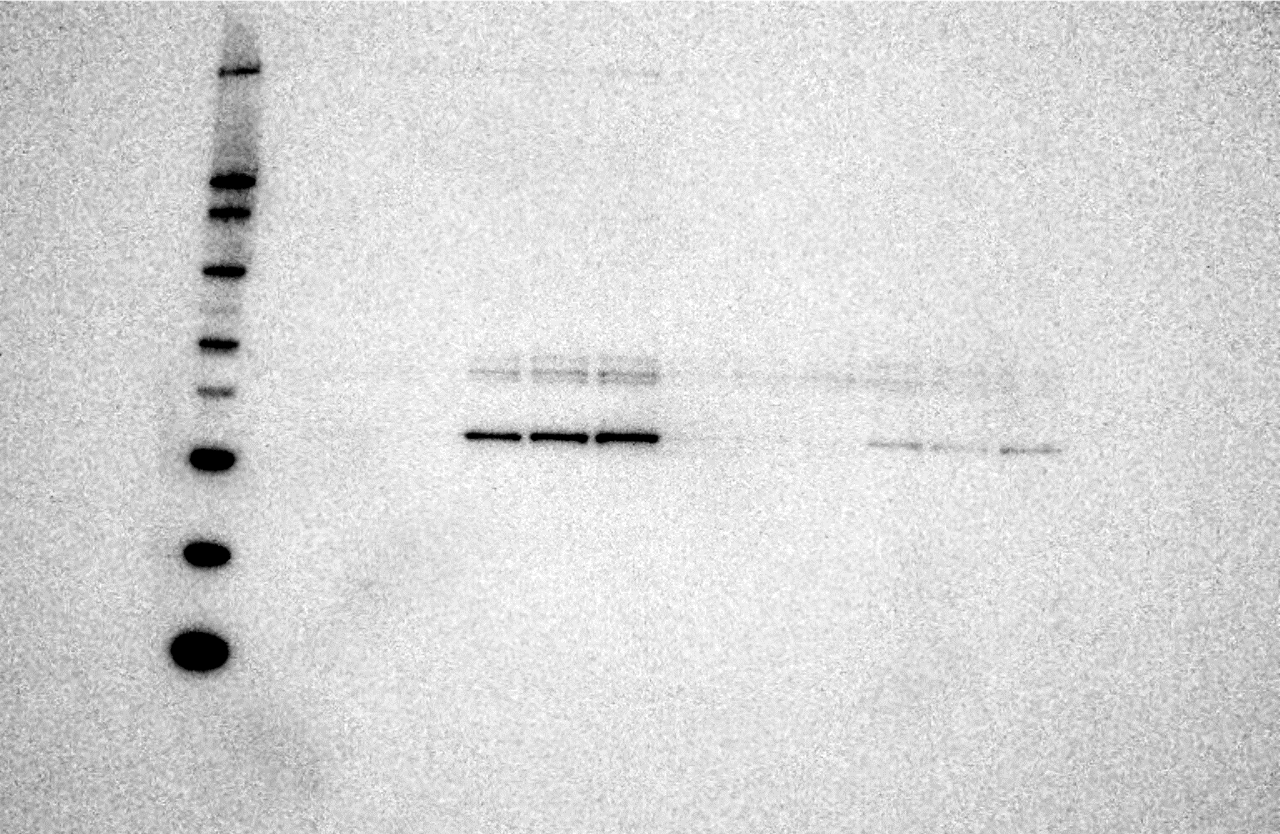

Supplement: Figure 5—source data 2. — Original blots for western blot analysis displayed in Figure 5. [file elife-98766-fig5-data2.zip › 5e-a-SMA.TIFF]

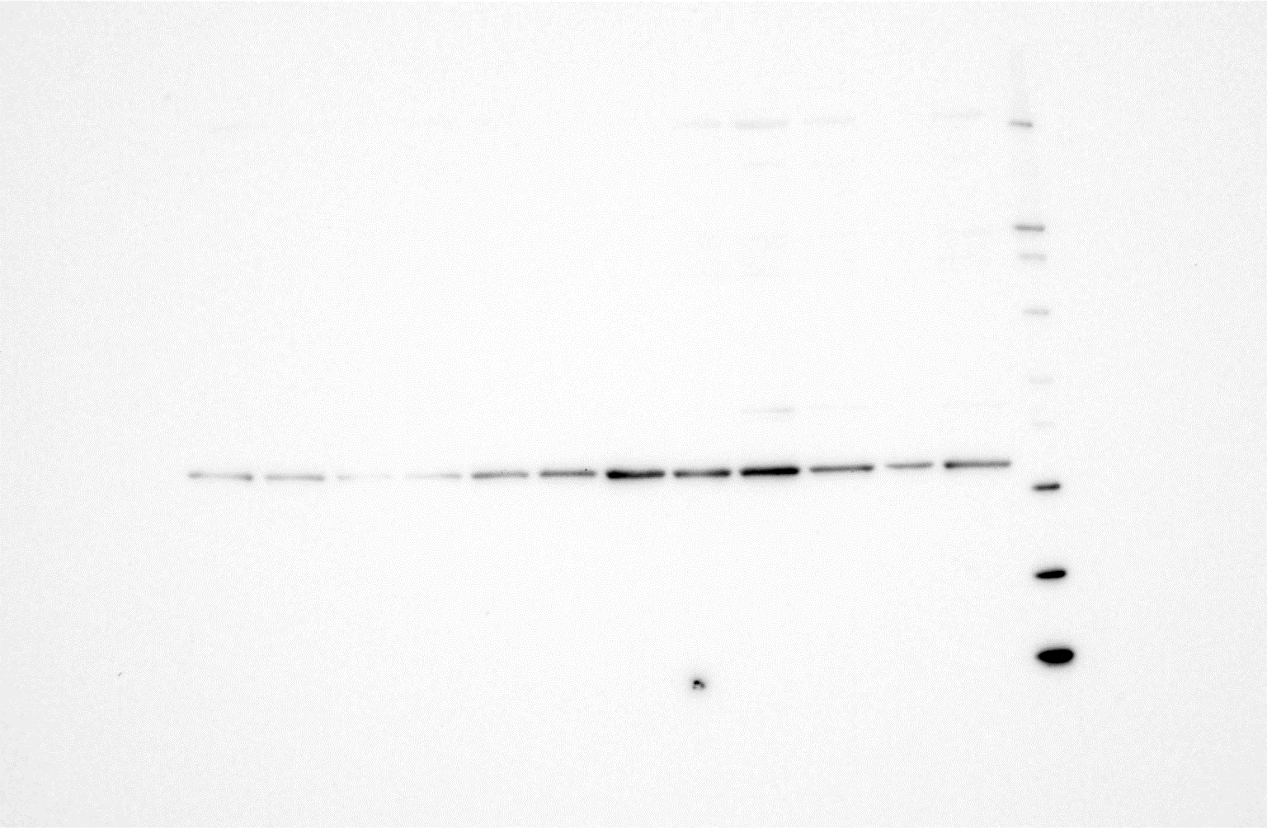

Supplement: Figure 5—source data 2. — Original blots for western blot analysis displayed in Figure 5. [file elife-98766-fig5-data2.zip › 5f a-SMA.TIFF]

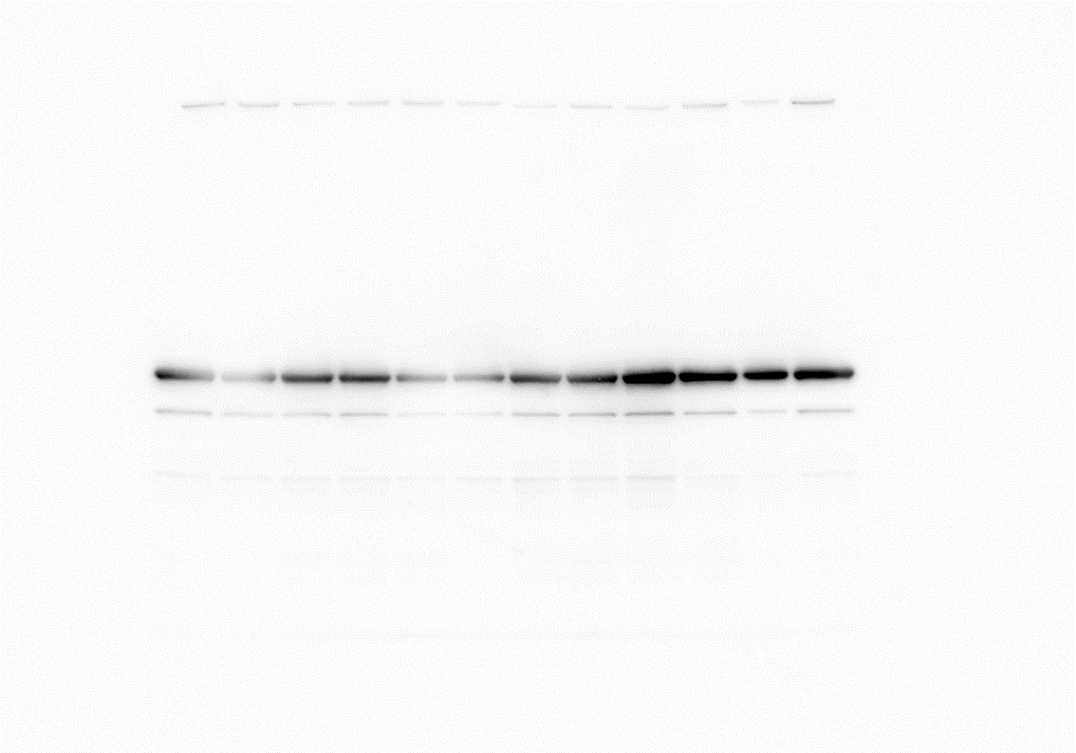

Supplement: Figure 5—source data 2. — Original blots for western blot analysis displayed in Figure 5. [file elife-98766-fig5-data2.zip › 5f a-tubulin.TIFF]

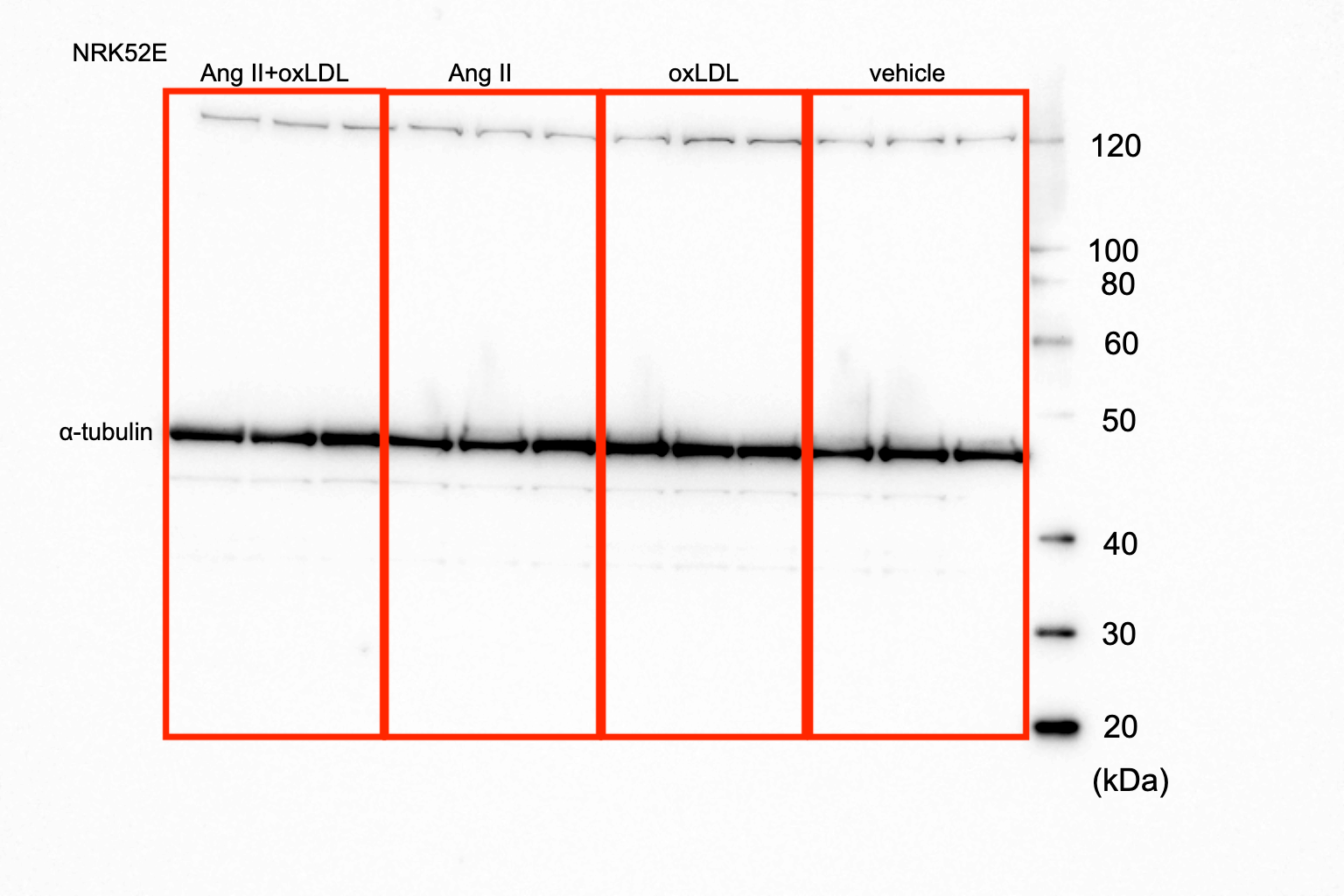

Supplement: Figure 5—source data 3. — Original western blots for Figure 5, indicating the relevant bands and treatments. [file elife-98766-fig5-data3.zip › 5d NRK52E a-tubulin AII+oxLDL.tiff]

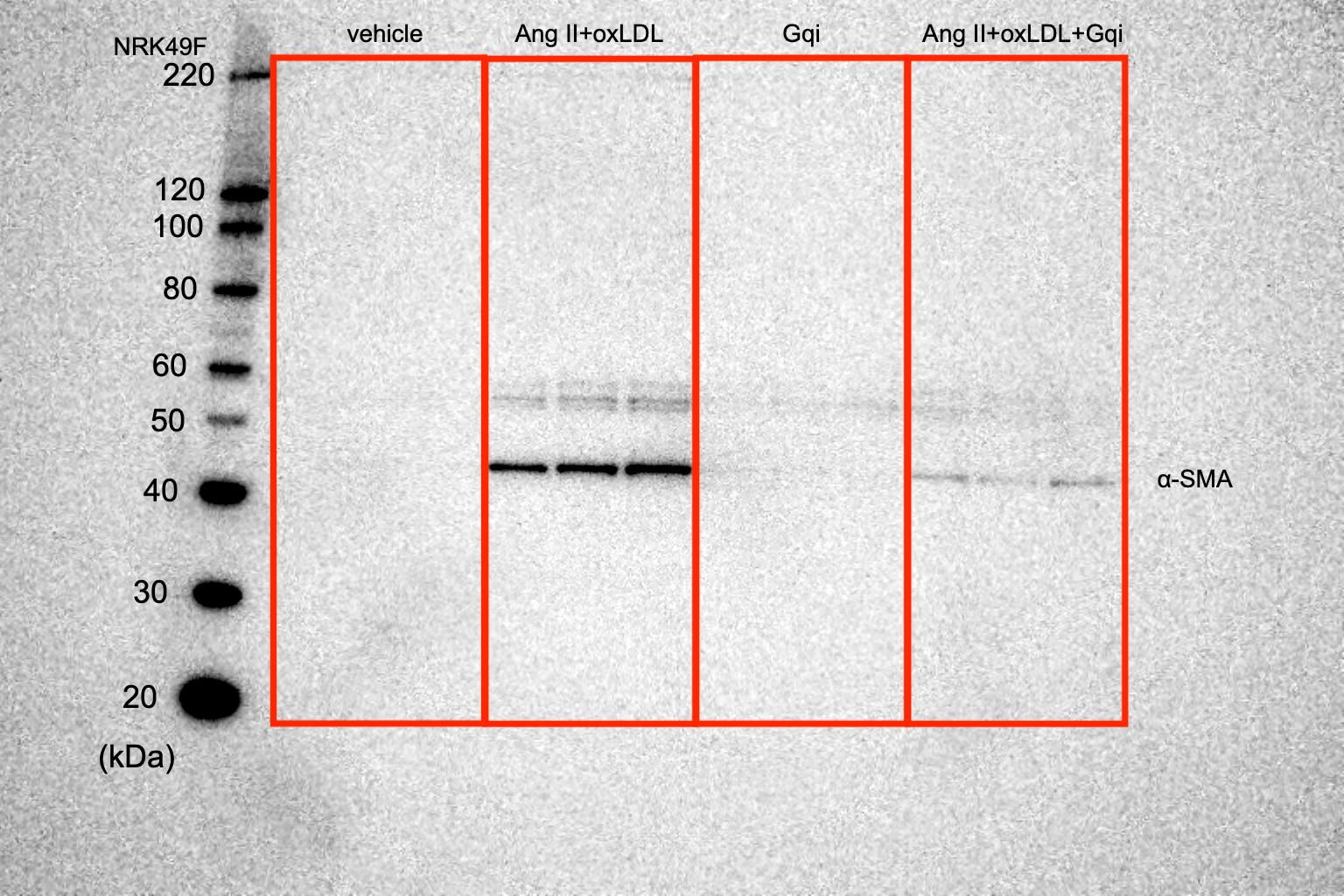

Supplement: Figure 5—source data 3. — Original western blots for Figure 5, indicating the relevant bands and treatments. [file elife-98766-fig5-data3.zip › 5e NRK49F a-SMA Gq.tiff]

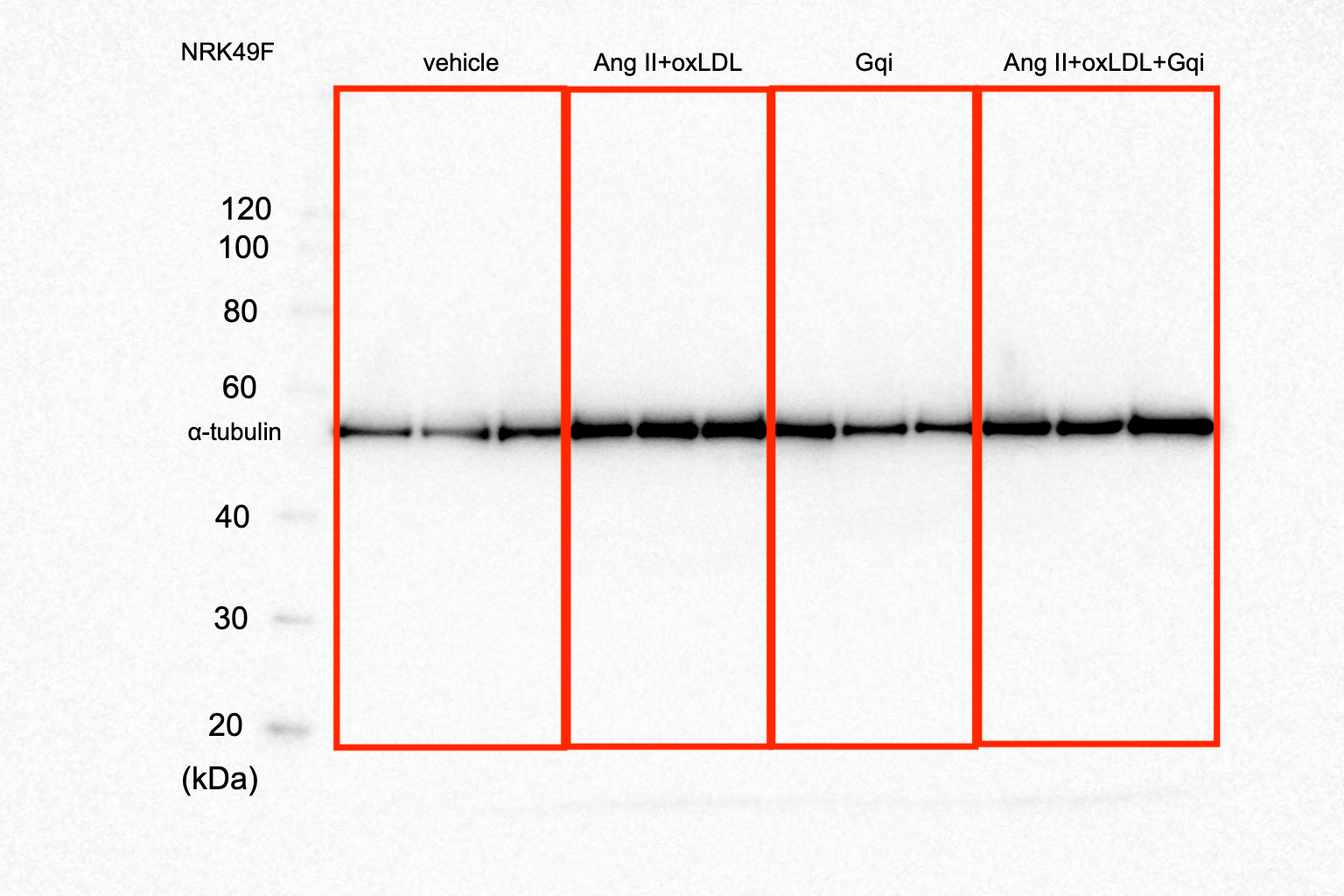

Supplement: Figure 5—source data 3. — Original western blots for Figure 5, indicating the relevant bands and treatments. [file elife-98766-fig5-data3.zip › 5e NRK49F a-tubulin Gq.tiff]

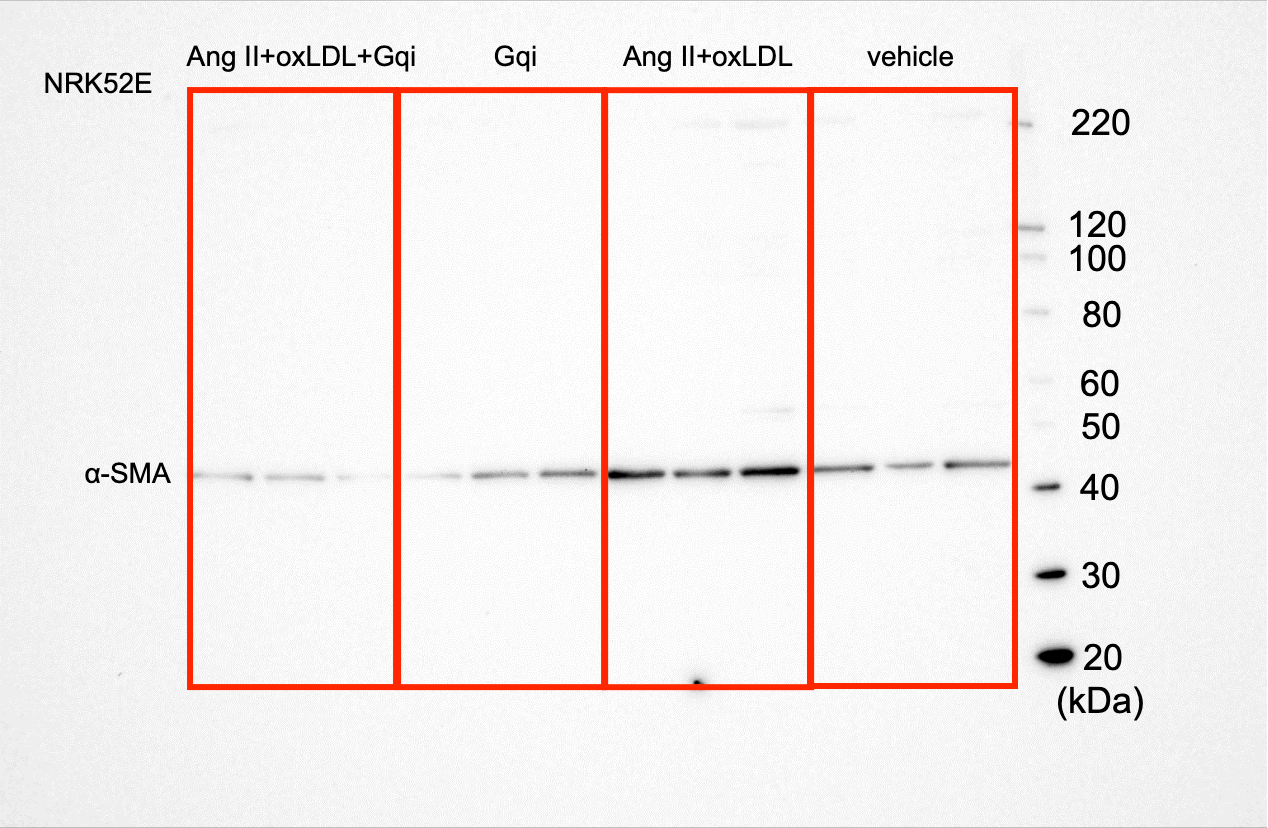

Supplement: Figure 5—source data 3. — Original western blots for Figure 5, indicating the relevant bands and treatments. [file elife-98766-fig5-data3.zip › 5f NRK52E a-SMA Gq.tiff]

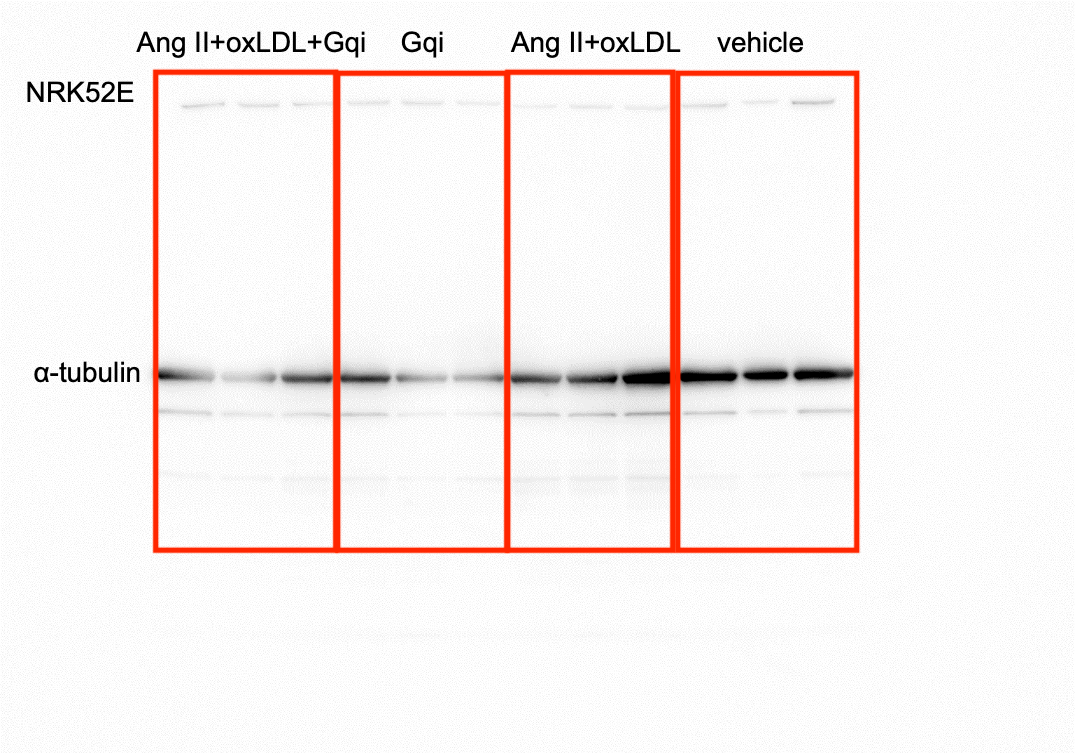

Supplement: Figure 5—source data 3. — Original western blots for Figure 5, indicating the relevant bands and treatments. [file elife-98766-fig5-data3.zip › 5f NRK52E a-tublin Gq.tiff]

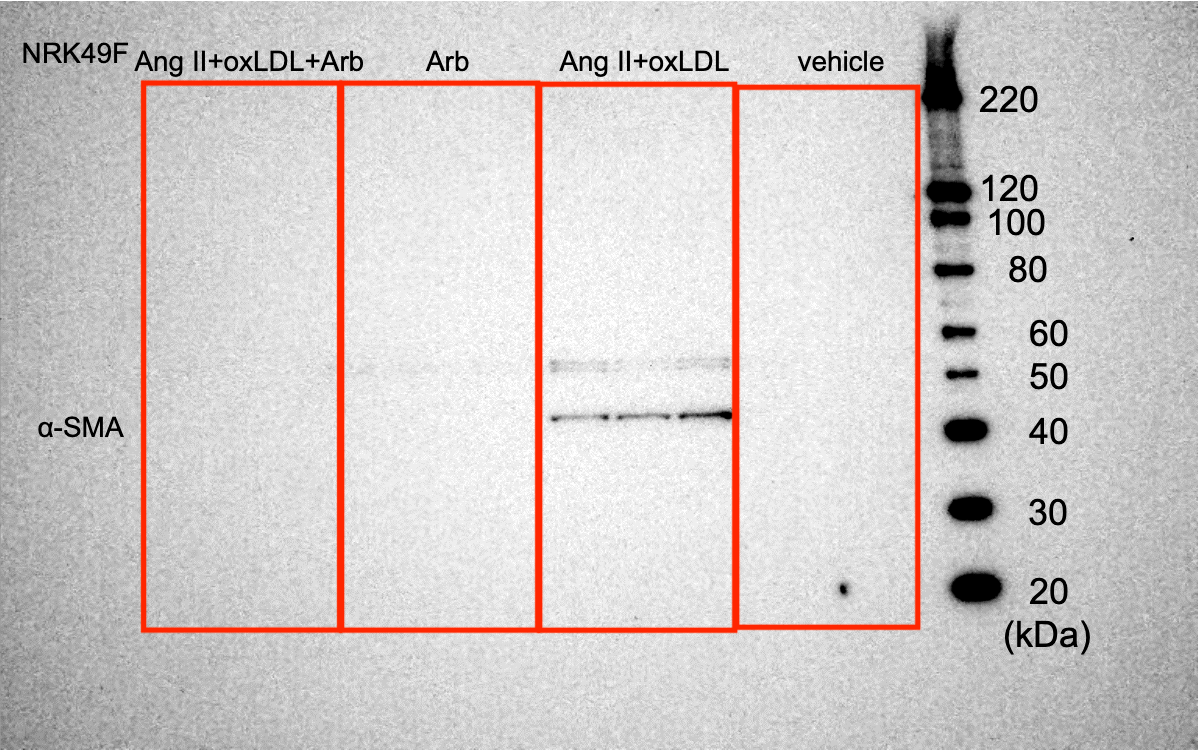

Supplement: Figure 5—source data 3. — Original western blots for Figure 5, indicating the relevant bands and treatments. [file elife-98766-fig5-data3.zip › 5g NRK49F a-sma Irbe.tiff]

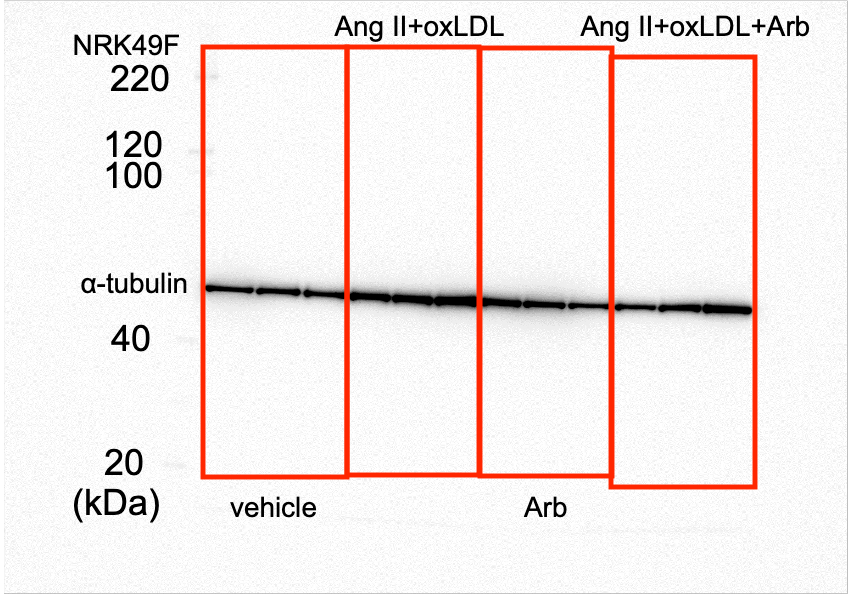

Supplement: Figure 5—source data 3. — Original western blots for Figure 5, indicating the relevant bands and treatments. [file elife-98766-fig5-data3.zip › 5g NRK49F a-tublin Irbe.tiff]

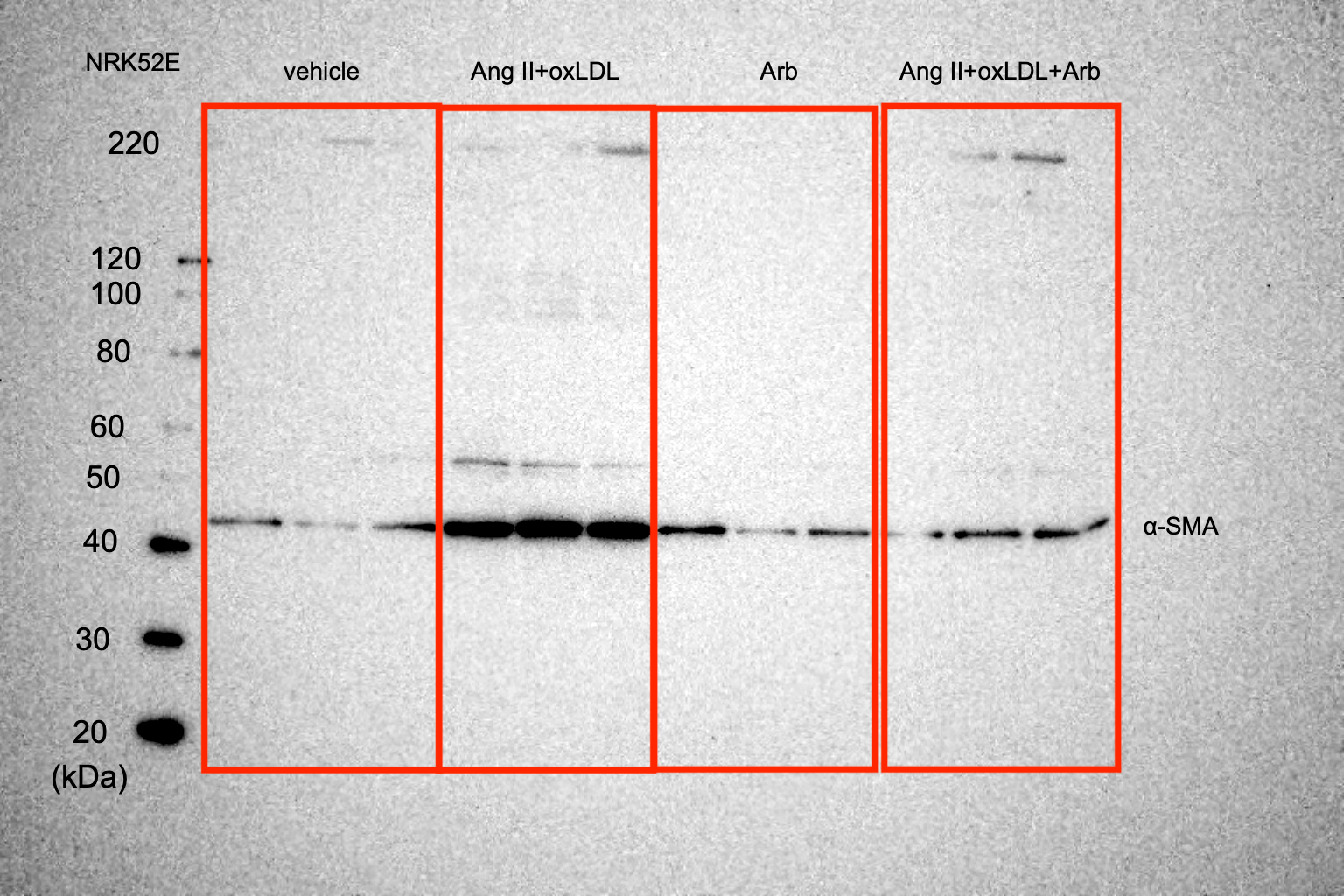

Supplement: Figure 5—source data 3. — Original western blots for Figure 5, indicating the relevant bands and treatments. [file elife-98766-fig5-data3.zip › 5h NRK52E a-SMA Irbe.tiff]

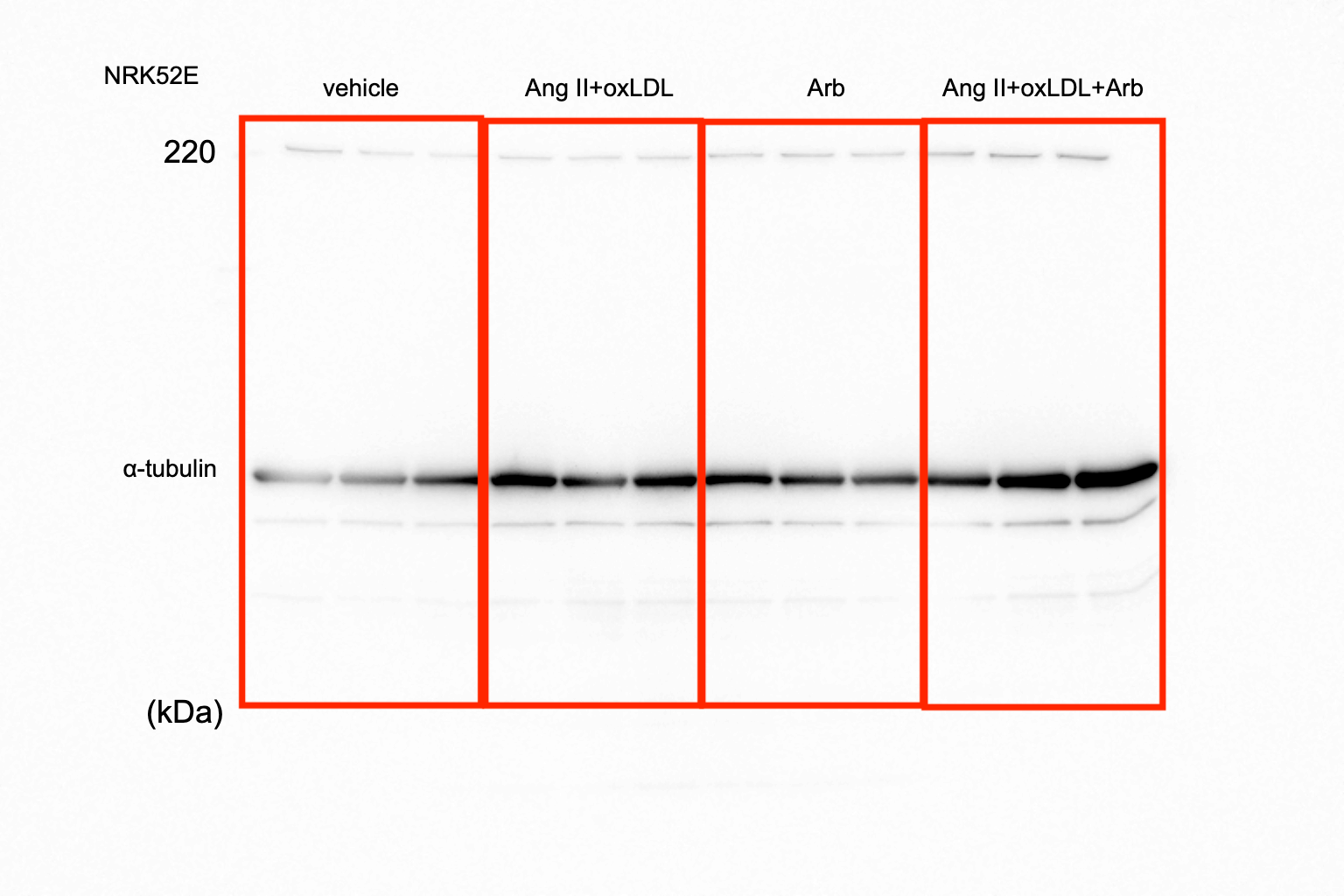

Supplement: Figure 5—source data 3. — Original western blots for Figure 5, indicating the relevant bands and treatments. [file elife-98766-fig5-data3.zip › 5h NRK52E a-tubulin Irbe.tiff]

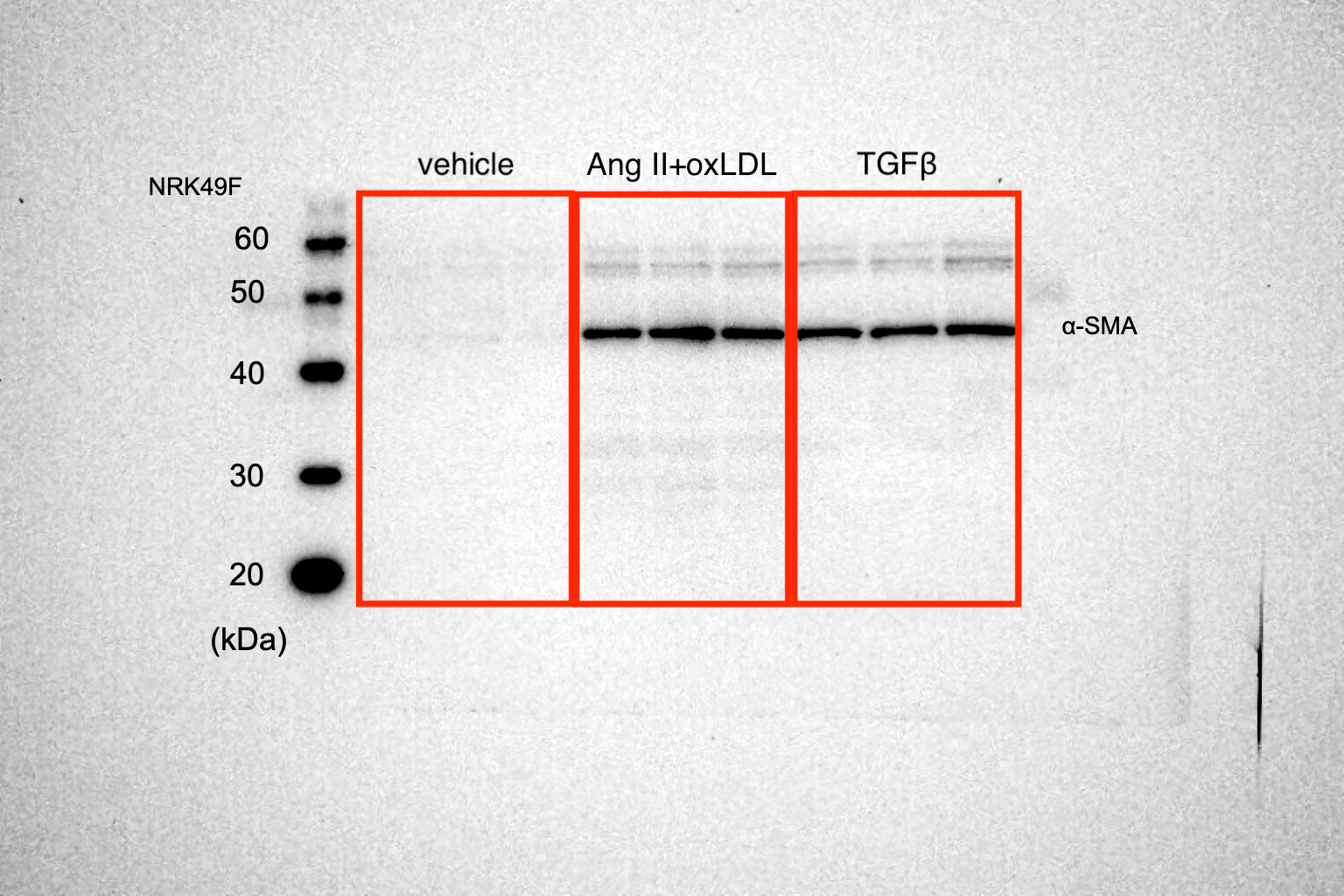

Supplement: Figure 5—source data 3. — Original western blots for Figure 5, indicating the relevant bands and treatments. [file elife-98766-fig5-data3.zip › 5a,a-SMA.tiff]

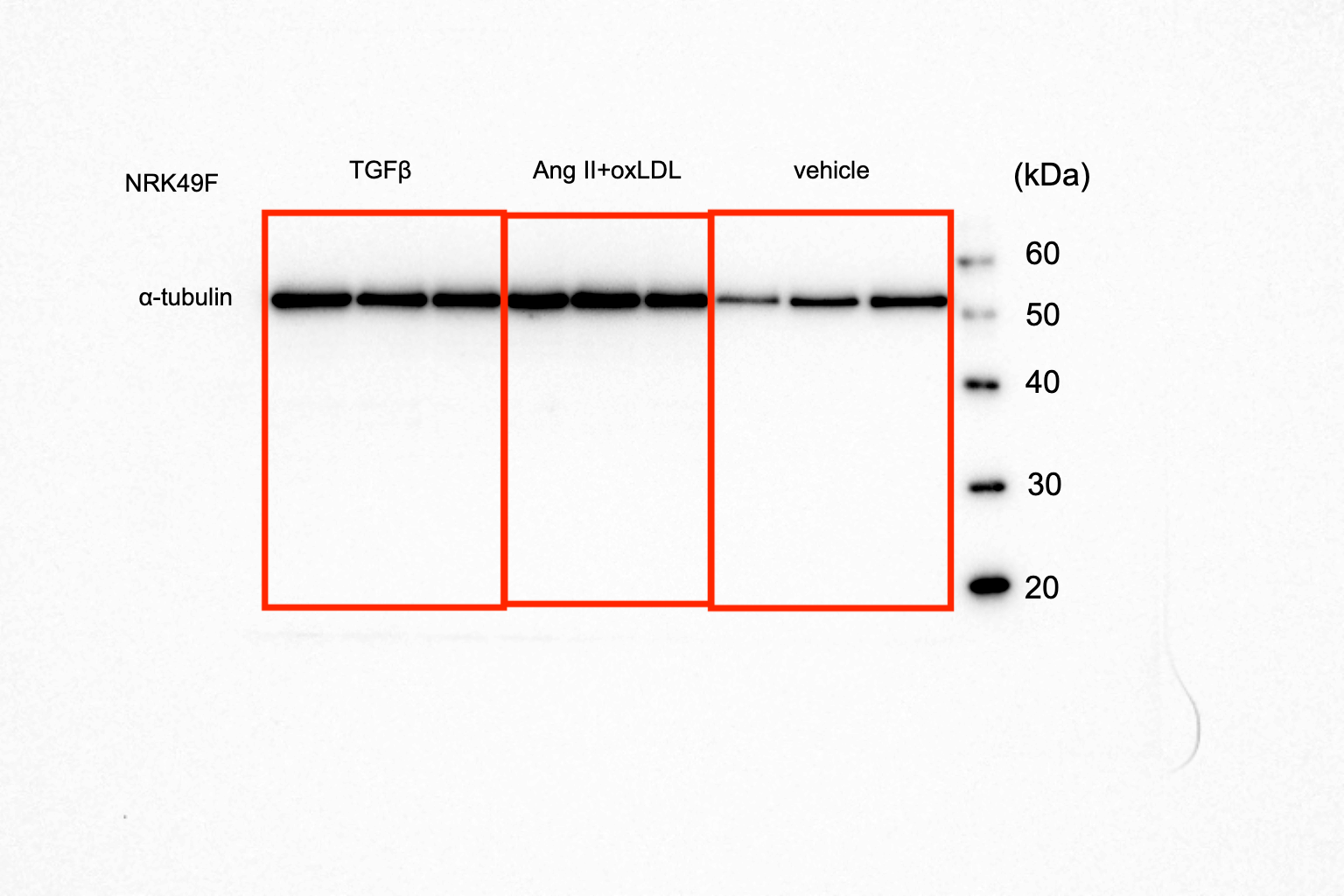

Supplement: Figure 5—source data 3. — Original western blots for Figure 5, indicating the relevant bands and treatments. [file elife-98766-fig5-data3.zip › 5a,a-tubulin.tiff]

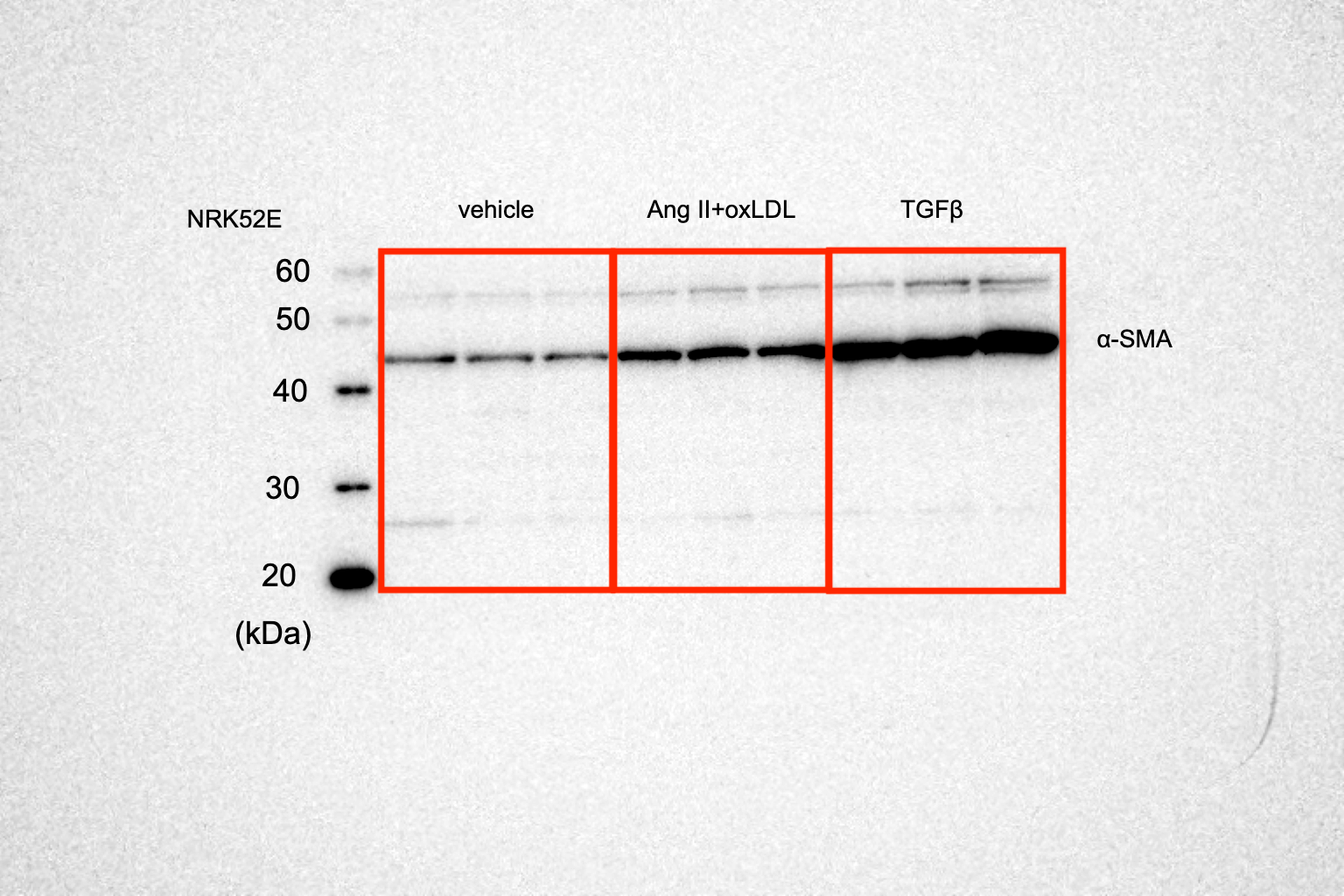

Supplement: Figure 5—source data 3. — Original western blots for Figure 5, indicating the relevant bands and treatments. [file elife-98766-fig5-data3.zip › 5b a-SMAt.tiff]

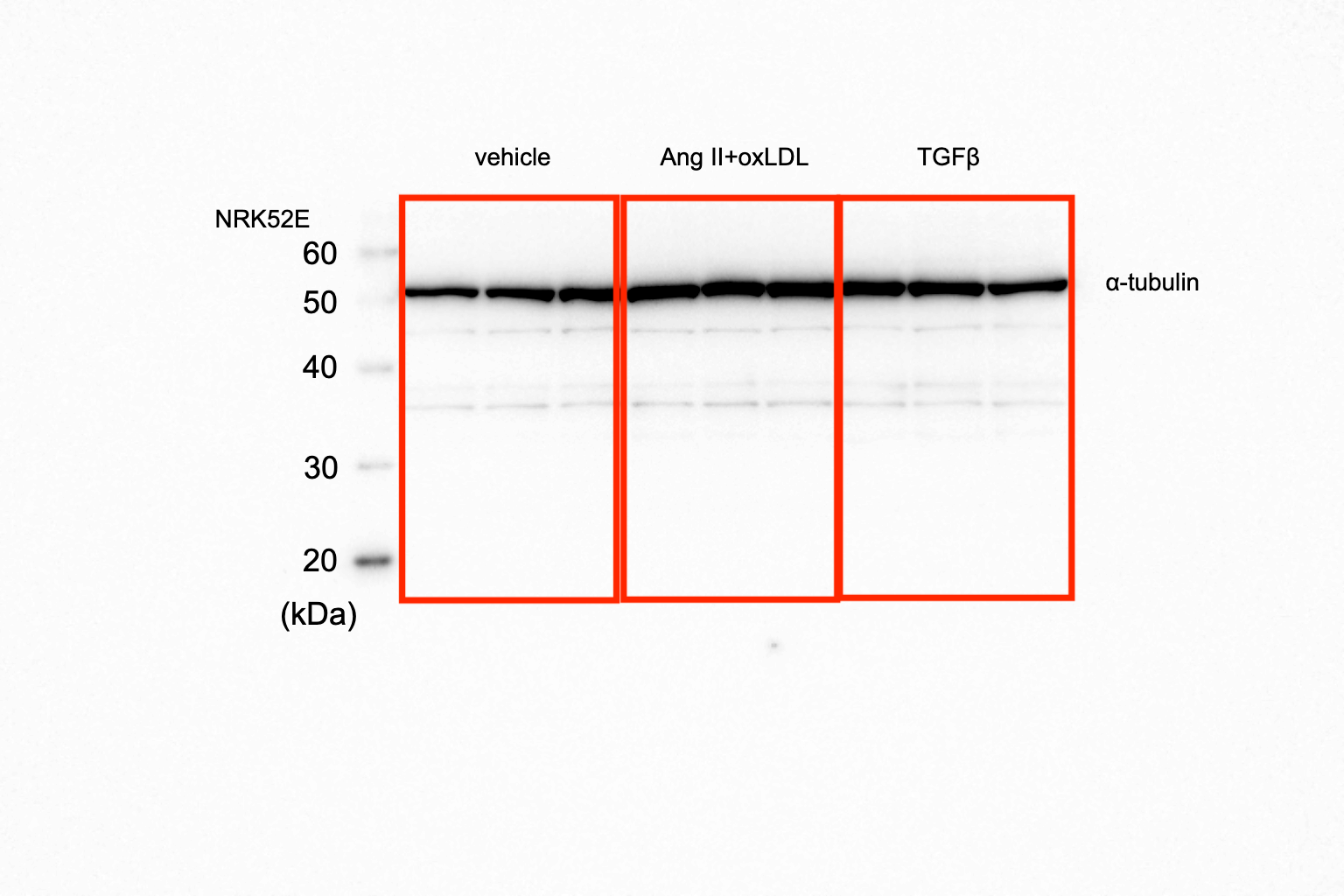

Supplement: Figure 5—source data 3. — Original western blots for Figure 5, indicating the relevant bands and treatments. [file elife-98766-fig5-data3.zip › 5b a-tubulin.tiff]

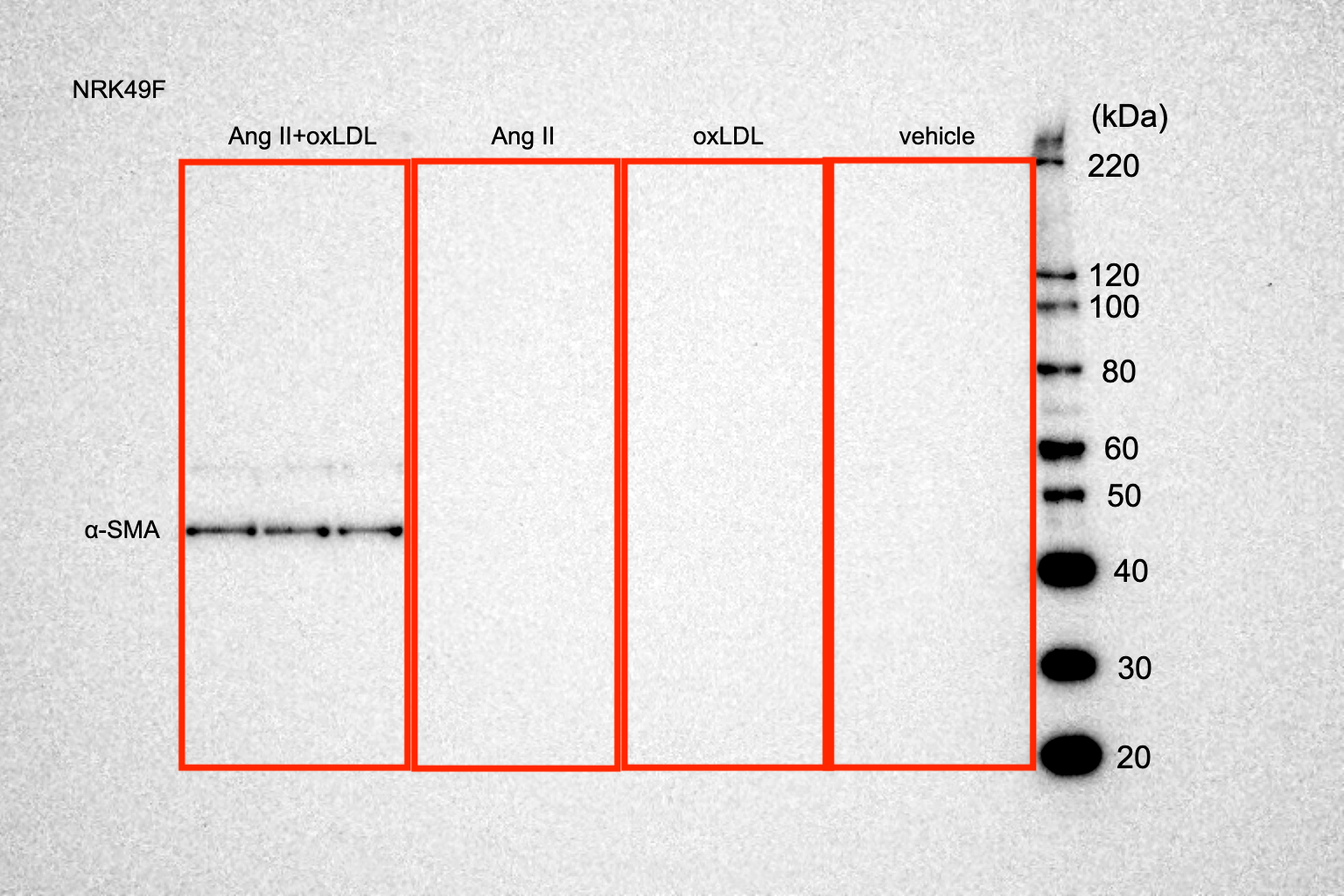

Supplement: Figure 5—source data 3. — Original western blots for Figure 5, indicating the relevant bands and treatments. [file elife-98766-fig5-data3.zip › 5c NRK49F a-SMA AII+oxLDL.tiff]

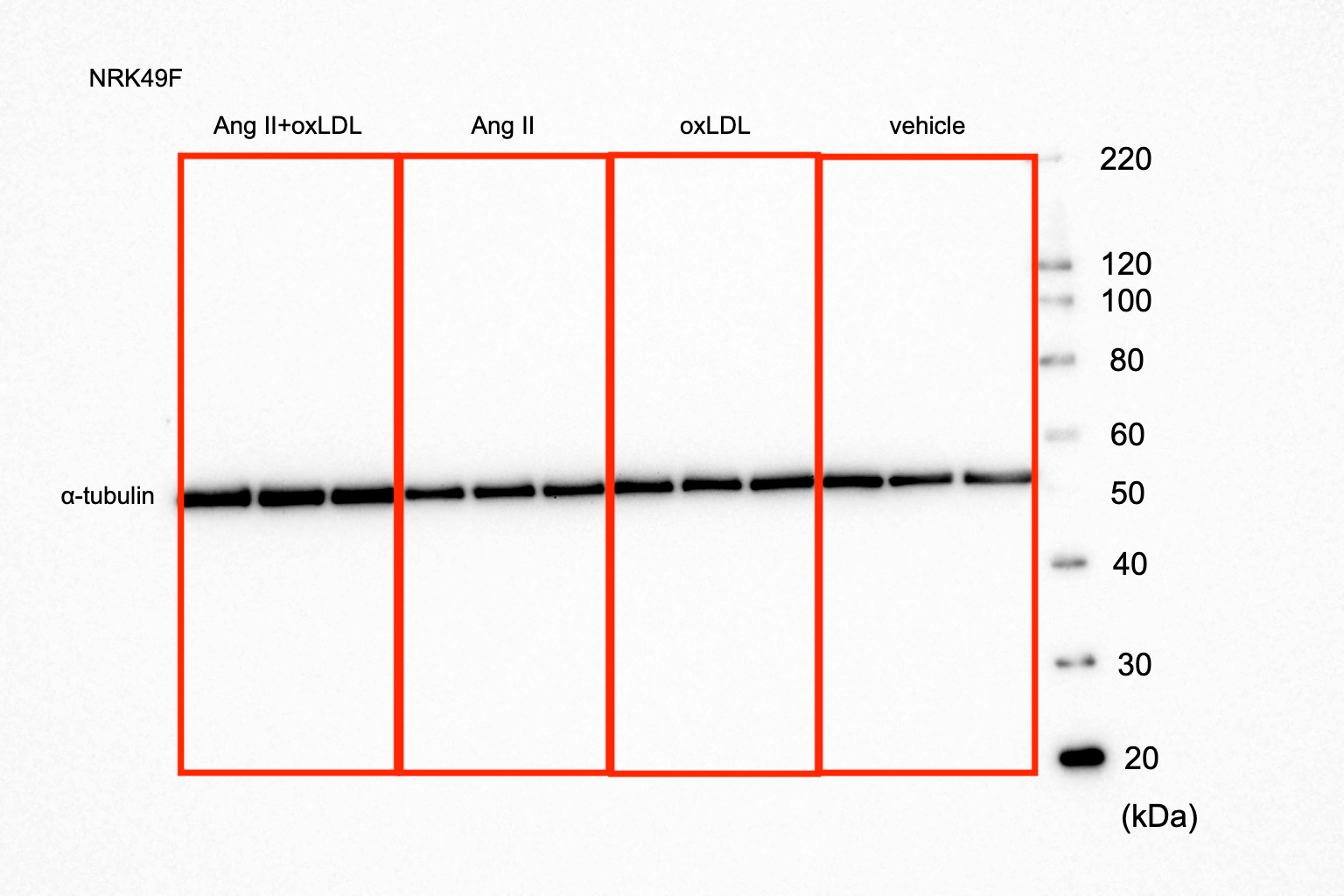

Supplement: Figure 5—source data 3. — Original western blots for Figure 5, indicating the relevant bands and treatments. [file elife-98766-fig5-data3.zip › 5c NRK49F a-tubulin AII+oxLDL.tiff]

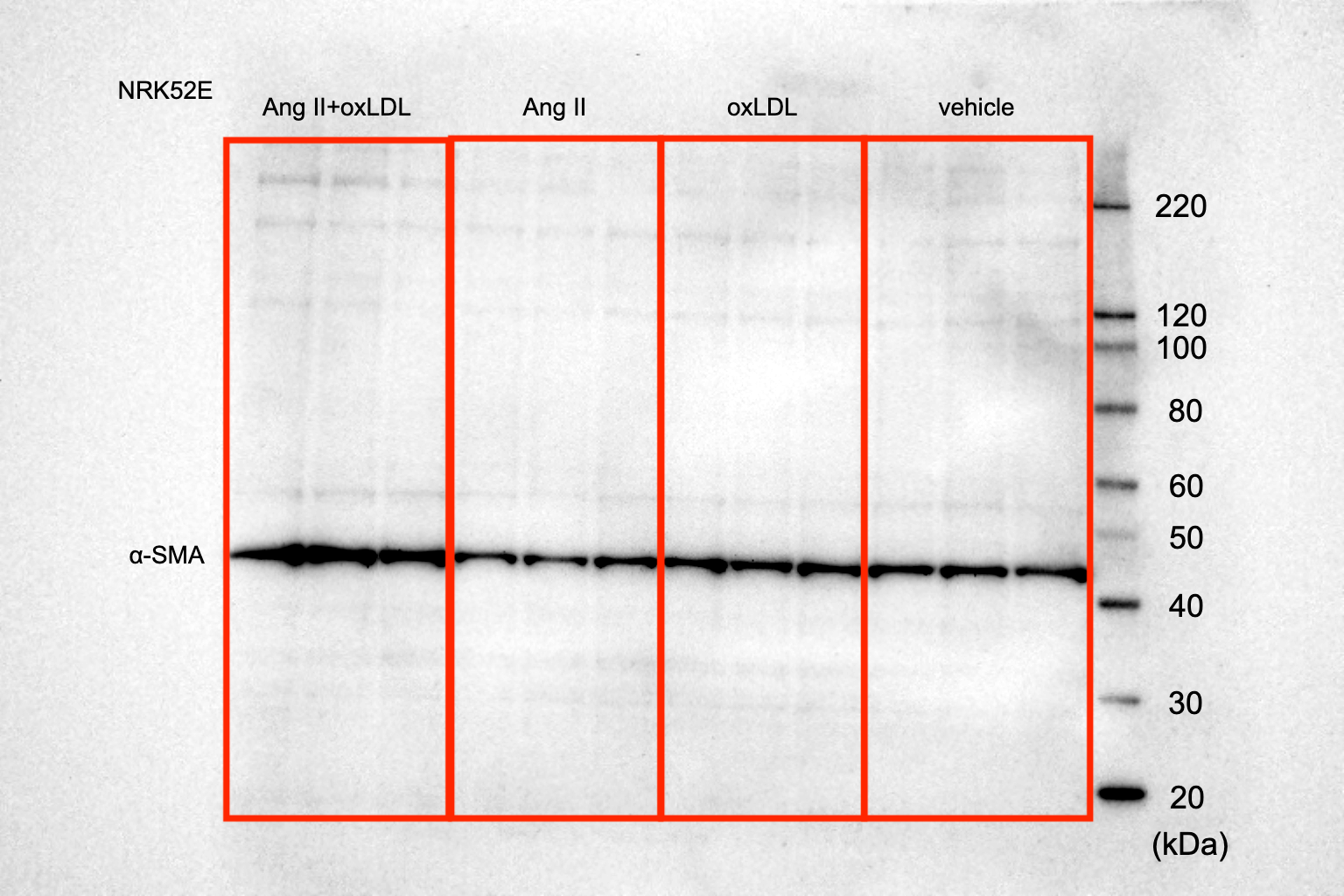

Supplement: Figure 5—source data 3. — Original western blots for Figure 5, indicating the relevant bands and treatments. [file elife-98766-fig5-data3.zip › 5d NRK52E a-SMA AII+oxLDL.tiff]
