# Supplementary material for: Oxidized low-density lipoprotein potentiates angiotensin II-induced Gq activation through the AT1-LOX1 receptor complex
Source: eLife. 2025 Mar 25;13:RP98766. doi: 10.7554/eLife.98766 (PMC11936421; doi:10.7554/eLife.98766)
Supplement: Supplementary file 1. — This table lists the gene symbols, names, and primer sequences used in the study. The first section contains primer sequences for rats, while the second section contains those for mice. [file elife-98766-supp1.docx]

Supplementary File 1. Primer sequences used in this study

Primer sequences for rats

| Symbol | Name | Forward (5`-3`) | Reverse (5`-3`) |
| --- | --- | --- | --- |
| *Gapdh* | glyceraldehyde-3-phosphate dehydrogenase | AGGAGTAAGAAACCCTGGAC | CTGGGATGGAATTGTGAG |
| *Tgfb2* | transforming growth factor, beta 2 | AGGAGGAATTTGGCCAGGTG | GCTCACGAGGAGGCTAATCC |
| *Tnf* | tumor necrosis factor | GGCATGGATCTCAAAGACAACC | AAATCGGCTGACGGTGTGG |
| *Il1b* | interleukin 1 beta | CACCTCTCAAGCAGAGCACAG | GGGTTCCATGGTGAAGTCAAC |
| *Il6* | interleukin 6 | GTCAACTCCATCTGCCCTTCAG | GGCAGTGGCTGTCAACAACAT |
| *Ccl2* | C-C motif chemokine ligand 2 | AGGCAGATGCAGTTAATGCCC | ACACCTGCTGCTGGTGATTCTC |
| *Ncf2* | neutrophil cytosolic factor 2 | AGCAGAAGAGCAGTTAGCATTGG | TGCTTTCCATGGCCTTGTC |
| *Cybb* | cytochrome b-245, beta polypeptide | TGGTGATGTTAGTGGGAGC | CTTTCTTGCATCTGGGTCT |
| *Fn1* | fibronectin 1 | AAAGCCAGCCCCTGGTTC | AGGTCACCTGTACCTGGAA |
| *Col1a1* | collagen, type I, alpha 1 | GACATCCCTGAAGTCAGCTGC | TCCCTTGGGTCCCTCGAC |
| *Col4a1* | collagen, type IV, alpha 1 | TGGCCTTGGAGGAAACTTTG | CTTGGAAACCTTGTGGACCAG |

Primer sequences for mice

| Symbol | Name | Forward (5`-3`) | Reverse (5`-3`) |
| --- | --- | --- | --- |
| *Gapdh* | glyceraldehyde-3-phosphate dehydrogenase | TTCCATCCTCCAGAAACCAG | CTCAGACCCCAGATCCAGAA |
| *Agtr1a* | angiotensin II receptor, type 1a | TGTCTGGCCGGAGAGGACT | TCTTTCATATGTTAAGTCCGGGAGA |
| *Agtr1b* | angiotensin II receptor, type 1b | GAGACCAGACAAGACACGCA | GTGAATTCAAAATGCACCCGT |
| *Olr1* | oxidized low density lipoprotein (lectin-like) receptor 1 | GGCCAACCATGGCTATGGGAGAATGG | CAGCGAACACAGCTCCGTCTTGAAGG |
| *Tgfb2* | transforming growth factor, beta 2 | TGATACGCCTGAGTGGCTGTCT | CACAAGAGCAGTGAGCGCTGAA |
| *Tnf* | tumor necrosis factor | GGCTGCCCCGACTACGT | ACTTTCTCCTGGTATGAGATAGCAAAT |
| *Il1b* | interleukin 1 beta | GTCACAAGAAACCATGGCACAT | GCCCATCAGAGGCAAGGA |
| *Il6* | interleukin 6 | CTGCAAGAGACTTCCATCCAGTT | AGGGAAGGCCGTGGTTGT |
| *Ccl2* | C-C motif chemokine ligand 2 | AAAACAGCATACATGGGAGACT | ATCCAGGGCACATATGCAGAG |
| *Ncf2* | neutrophil cytosolic factor 2 | CTGGCTGAGGCCATCAGACT | AGGCCACTGCAGAGTGCTTG |
| *Cybb* | cytochrome b-245, beta polypeptide | TTGGGTCAGCACTGGCTCTG | TGGCGGTGTGCAGTGCTATC |
| *Ncf4* | neutrophil cytosolic factor 4 | GCCGCTATCGCCAGTTCTAC | GCAGGCTCAGGAGGTTCTTC |
| *Ncf1* | neutrophil cytosolic factor 1 | GATGTTCCCCATTGAGGCCG | GTTTCAGGTCATCAGGCCGC |
| *Fn1* | fibronectin 1 | AAGACCATACCTGCCGAATG | GAACATGACCGATTTGGACC |
| *Col1a1* | collagen, type I, alpha 1 | GAAGCACGTCTGGTTTGGA | ACTCGAACGGGAATCCATC |
| *Col4a1* | collagen, type IV, alpha 1 | CCAAAAGGACAGCAAGGTGTG | CCAGGAAACCCTCTTGGACC |
| *Acta2* | actin alpha 2, smooth muscle, aorta | GACGTACAACTGGTATTGTG | TCAGGATCTTCATGAGGTAG |
| *Vim* | vimentin | AGCTGCTAACTACCAGGACACTATTG | CGAAGGTGACGAGCCATCTC |

Primer sequences for mice (cont’d)

| Symbol | Name | Forward (5`-3`) | Reverse (5`-3`) |
| --- | --- | --- | --- |
| *Cdh1* | cadherin 1 | GTCTCCTCATGGCTTTGC | CTTTAGATGCCGCTTCAC |
| *Cdh16* | cadherin 16 | CTGGCAGCGATAGGCTTCAT | TGGGGCTGCTTGGATCATTC |
| *Lcn2* | lipocalin 2 | TCTGTCCCCACCGACCAA | GGAAAGATGGAGTGGCAGACA |
| *Ptgs2* | prostaglandin-endoperoxide synthase 2 | TGGTGAAAACTGTACTACACCTGA | CTTCGCAGGAAGGGGATGTT |
